# Decreased Risk of Ventilator-Associated Pneumonia in Sepsis Due to Intra-Abdominal Infection

**DOI:** 10.1371/journal.pone.0137262

**Published:** 2015-09-04

**Authors:** François Philippart, Gaëlle Bouroche, Jean-François Timsit, Maité Garrouste-Orgeas, Elie Azoulay, Michael Darmon, Christophe Adrie, Bernard Allaouchiche, Claire Ara-Somohano, Stéphane Ruckly, Anne-Sylvie Dumenil, Bertrand Souweine, Dany Goldgran-Toledano, Lila Bouadma, Benoît Misset, Outcomerea study group

**Affiliations:** 1 Medical-Surgical ICU, Groupe Hospitalier Paris Saint Joseph, Paris, France; 2 Department of Anesthesia and Intensive Care, Gustave Roussy Institute, Villejuif, France; 3 Université Grenoble 1, U823, Albert Bonniot Institute, La Tronche, France; 4 Medical ICU, Groupe hospitalier Bichat-Claude Bernard, Paris, France; 5 Medical ICU, Saint Louis Teaching Hospital, Paris, France; 6 Université Paris VII—Denis Diderot, Paris, France; 7 Medical ICU, St Etienne Hospital, St Etienne, France; 8 Physiology laboratory, Cochin hospital, Paris, France; 9 Surgical ICU, Edouart Herriot Hospital, Lyon, France; 10 Université Lyon I—Claude Bernard, Lyon, France; 11 Medical ICU, Albert Michallon Teaching Hospital, Grenoble, France; 12 Surgical ICU, Antoine Béclère Hospital, Clamart, France; 13 Medical ICU, Gabriel Montpied University Hospital, Clermont-Ferrand, France; 14 Medical-Surgical ICU, Gonesse Hospital, Gonesse, France; 15 Université Paris Descartes, Paris, France; University of Pittsburgh, UNITED STATES

## Abstract

**Rationale:**

Experimental studies suggest that intra-abdominal infection (IAI) induces biological alterations that may affect the risk of lung infection.

**Objectives:**

To investigate the potential effect of IAI at ICU admission on the subsequent occurrence of ventilator-associated pneumonia (VAP).

**Methods:**

We used data entered into the French prospective multicenter Outcomerea database in 1997–2011. Consecutive patients who had severe sepsis and/or septic shock at ICU admission and required mechanical ventilation for more than 3 days were included. Patients with acute pancreatitis were not included.

**Measurements and Main Results:**

Of 2623 database patients meeting the inclusion criteria, 290 (11.1%) had IAI and 2333 (88.9%) had other infections. The IAI group had fewer patients with VAP (56 [19.3%] *vs*. 806 [34.5%], *P*<0.01) and longer time to VAP (5.0 *vs*.10.5 days; *P*<0.01). After adjustment on independent risk factors for VAP and previous antimicrobial use, IAI was associated with a decreased risk of VAP (hazard ratio, 0.62; 95% confidence interval, 0.46–0.83; *P*<0.0017). The pathogens responsible for VAP were not different between the groups with and without IAI (*Pseudomonas aeruginosa*, 345 [42.8%] and 24 [42.8%]; *Enterobacteriaceae*, 264 [32.8%] and 19 [34.0%]; and *Staphylococcus aureus*, 215 [26.7%] and 17 [30.4%], respectively). Crude ICU mortality was not different between the groups with and without IAI (81 [27.9%] and 747 [32.0%], *P* = 0.16).

**Conclusions:**

In our observational study of mechanically ventilated ICU patients with severe sepsis and/or septic shock, VAP occurred less often and later in the group with IAIs compared to the group with infections at other sites.

## Introduction

Despite the availability of numerous therapeutic interventions, ventilator-acquired pneumonia (VAP) remains a major public health issue [1]. VAP is the leading nosocomial infection in intensive care unit (ICU) patients and is responsible for high morbidity and mortality rates [2]. Risk factors for VAP include patient characteristics such as co-morbidities and immunodeficiency due, for instance, to chronic corticosteroid therapy or AIDS. In ICU patients, acute alterations in innate and adaptative immunity with immunoparalysis characterized by impaired antigen presentation and endotoxin tolerance may play a pivotal role in the occurrence of nosocomial infections [3, 4]. Endotoxin tolerance is due to a substantial decrease in the cytokine response to lipopolysaccharide (LPS) after the first LPS stimulation, as shown in both animal models and humans [5, 6]. In patients with sepsis, greater magnitude [7] and longer duration [8] of endotoxin tolerance are associated with adverse outcomes. However, the role for endotoxin tolerance in the pathophysiology of nosocomial infections remains unclear. Recent studies suggest that endotoxin tolerance may involve re-programming of immune cells [9] responsible for changes in their behavior without impairments in phagocytic [10], or bactericidal capabilities [10].

In patients with peritonitis, large amounts of LPS are found both within the peritoneal cavity, and in the lymph and blood (especially in the hepatic circulation) [11]. Whether the resulting immune cell exposure to LPS influences the risk of VAP in ICU patients is unknown. In animal models, LPS exposure or peritoneal bacterial infection induced by a single local *Escherichia coli* injection or by cecal ligation and puncture is usually associated with better infection control during bacteremia or a second intra-abdominal infection (IAI), with improved bacterial clearance as the underlying mechanism [12–14]. Nonetheless, these models have produced conflicting results in terms of control of subsequent respiratory infection [13, 15, 16]. In some models, peritonitis was associated with worse outcomes of Gram-positive or Gram-negative pneumonia [15, 16], whereas another study suggested increased clearance of *Pseudomonas aeruginosa* [13]. Although emergency surgery is associated with an increased risk of nosocomial infection [17], whether IAI affects the risk of subsequent respiratory tract infection is unknown.

To investigate the potential influence of IAI on the risk of subsequent respiratory infection, we compared the incidence of VAP and outcomes in patients admitted to the ICU with severe sepsis and/or septic shock due to IAI versus infections at other sites. To this end, we used the multicenter prospective database Outcomerea [2].

## Material and Methods

### Study population

We used all the records in the French multicenter prospective database Outcomerea covering the 15-year period from 1997 to 2011. Data were collected as previously described [2]. Briefly, participating ICUs provided a random sample of at least 50 ICU stays longer than 24 hours. For each patient, admission characteristics and in-ICU events and scores were recorded daily. The following were recorded at admission: demographic data, admission diagnosis and admission category (medical, scheduled surgery, or emergent surgery), chronic co-morbidities (using the Knaus [18] definition and including the McCabe [19] score), clinical findings, and laboratory test results. The Simplified Acute Physiology Score (SAPS) II [20] and Sequential Organ Failure Assessment (SOFA) [21] were computed at admission then once a day. In addition, data on procedures and treatments were collected: antibiotics, enteral feeding, corticosteroids in doses greater than 0.5 mg/Kg, invasive or noninvasive mechanical ventilation, vasopressors, hemodialysis, insertion and presence of invasive devices (arterial catheter, central venous catheter, Swan-Ganz catheter, and Foley catheter), tracheotomy, and do-not-resuscitate orders. Hereafter, we refer to the above-described variables as severity-of-illness indicators.

Inclusion criteria were age over 18 years, mechanical ventilation started at ICU admission and continued for more than 3 calendar-days, and severe sepsis or septic shock. We did not include patients with acute pancreatitis.

### Definitions and patient groups

IAI was defined as any of the following diagnoses: primary peritoneal abscess, secondary or postoperative peritonitis, and acute cholangitis. Because of their retroperitoneal location and non portal blood drainage, urinary tract infections were not classified as IAIs. We used this definition to classify the patient into two groups, with and without IAI infection, respectively.

VAP was defined as new and persistent pulmonary infiltrates on chest radiographs combined with purulent tracheal secretions and/or body temperature greater than or equal to 38.5°C or less than or equal to 36.5°C and/or blood leukocyte count greater than or equal to 10·10^9^/L. A definitive diagnosis of VAP required microbiological confirmation by quantitative culture of a protected specimen brush (>10^3^ cfu/mL), bronchoalveolar lavage (BAL) fluid specimen (>10^4^cfu/mL), or endotracheal aspirate (>10^5^ cfu/mL) [22]. Severe sepsis was defined as the presence of acute organ dysfunction secondary to infection and septic shock as severe sepsis with hypotension requiring vasopressor therapy.

Bacterial resistance was defined according to the bacterial species: resistance to methicillin for *Staphylococcus aureus*; resistance to ticarcillin, ceftazidime, or imipenem for *P*. *aeruginosa*; and extended-spectrum beta-lactamase production or cephalosporinase hyperproduction for *Enterobacteriaceae*.

### Ethical issues

The collect and use of data from the Outcomerea database was approved by the institutional review board of the Outcomerea leader (CECIC Clermont-Ferrand—IRB n°5891; Ref: 2007–16), which waived the need for signed informed consent of the participants, in accordance with French legislation on non-interventional studies. However, the patients and their next of kin were asked whether they were willing to participate in the database, and none declined participation.

### Statistical analysis

Qualitative and quantitative patient characteristics described as number (%) or median (interquartile range [IQR]), respectively, were compared between the groups with and without IAI using the chi-square or Mann-Whitney tests, as appropriate. Cumulative incidence curves of the risk of VAP and of death were plotted for each group. Endpoints (VAP and death) were censored on day 30. Patients discharged from the ICU (to their home or to an extended-care facility) were checked to be free of VAP and alive on day 30.

To estimate the impact of IAI on the risk of VAP, we used a Cox model [23] with VAP as the dependent variable. Antibiotic use was handled as a time-dependent variable assigned a value of 1 if present on the previous day and a value of 0 otherwise. We then used stepwise selection to adjust the impact of IAI for confounding factors. The results are reported as hazard ratios (HRs) and 95% confidence intervals (95%CIs) with ICU day 3 defined as time 0. Data were censored on day 30.

We modeled the risk of day-30 mortality after VAP using another Cox model in the subgroup with VAP. Parameters associated with death after VAP were selected using a bivariate model. The impact of IAI on day-30 VAP-associated mortality was assessed using the same model, with or without adjustment on other prognostic co-variates. The results are reported as HRs and 95% CIs with the day of VAP onset defined as time 0.

*P* values <0.05 were considered significant. Analyses were computed using SAS 9.2software (SAS Institute; Cary, NC).

## Results

### Descriptive data

**Fig 1:**
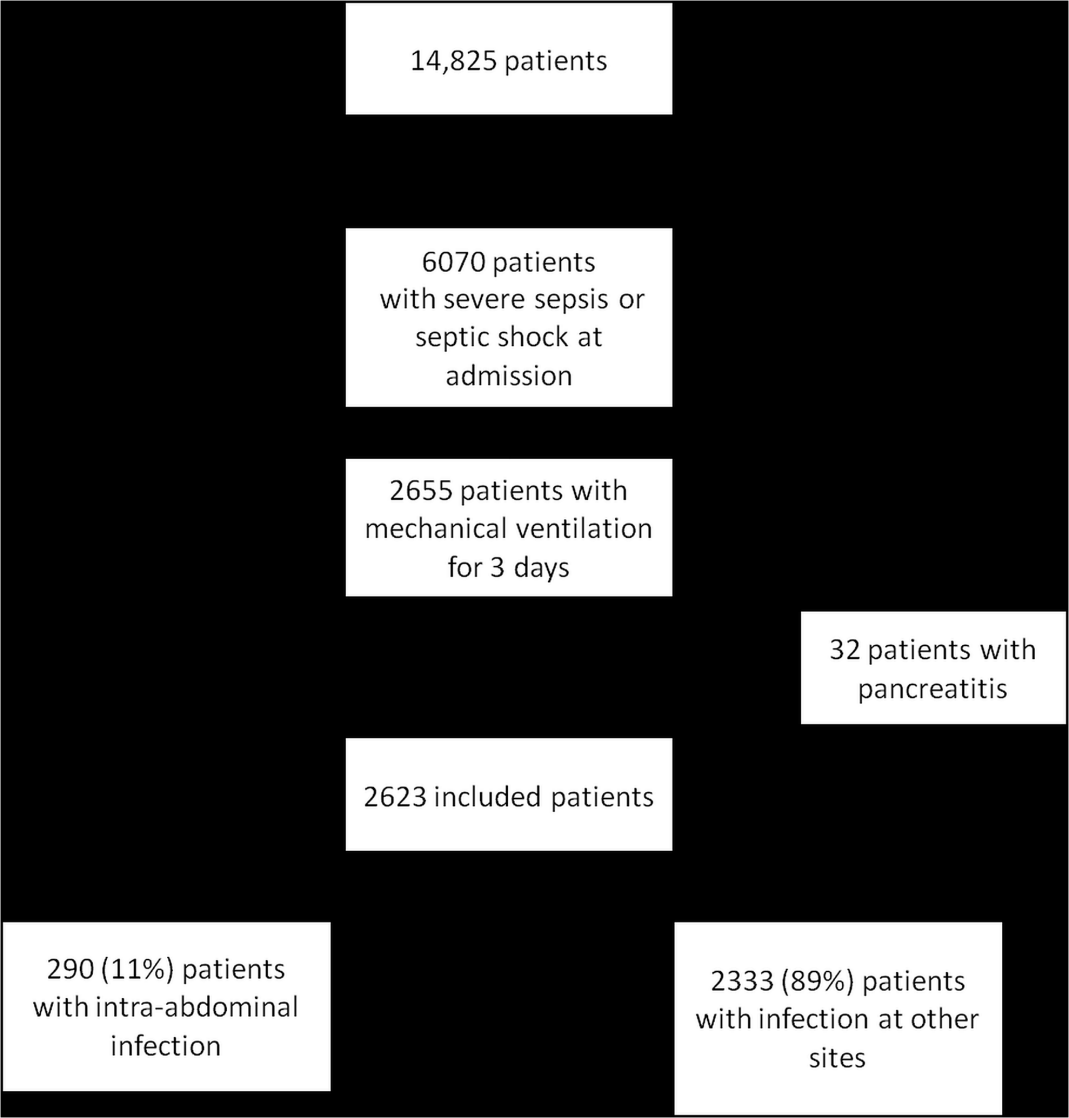
Flow Chart.

Of 14,825 patients in the Outcomerea database, 2623 fulfilled the inclusion criteria (mechanical ventilation >3 days and severe sepsis and/or septic shock) and did not have pancreatitis. Among them, 290 (11.1%) had IAI at ICU admission and 2333 (88.9%) had infections at other sites (Fig 1).

The main reasons for ICU admission were septic shock in the IAI group (60.3%) and acute respiratory failure (38.8%), septic shock (18%), and coma (17%) in the non-IAI group. Most IAI patients (77.2%) were admitted for emergent surgery and most non-IAI patients (80.0%) for medical reasons. The two groups had similar acute-illness severity as reflected by the SAPS II and SOFA score (Table 1).

**Table 1:** Patient characteristics.

	N (%)		
variable	Patients without IAI	Patients with IAI	*P* value
	(n = 2333)	(n = 290)	
Age in y, median (IQR)	66 [54; 76]	71 [60; 78]	<.01
Male	1510 (64.7)	158 (54.5)	<.01
Smoking habits	574 (24.6)	46 (15.9)	<.01
BMI, median (IQR)	25.3 [22.5; 27.8]	25.3 [23; 29.1]	0.05
Main reason for ICU admission			
Coma	396 (17.0)	8 (2.8)	<.01
Respiratory distress	905 (38.8)	34 (11.7)	<.01
Septic shock	421 (18.0)	175 (60.3)	<.01
Hemorrhagic shock	73 (3.1)	5 (1.7)	0.18
Cardiogenic shock	105 (4.5)	1 (0.3)	<.01
Other shock	65 (2.8)	8 (2.8)	0.98
MOF	79 (3.4)	32 (11.0)	<.01
Trauma	20 (0.9)	3 (1.0)	0.76
COPD symptoms	106 (4.5)	3 (1.0)	<.01
Admission category			<.01
Medical	1899 (80.0)	39 (13.5)	
Scheduled surgery	196 (8.4)	27 (9.3)	
Emergency surgery	271 (11.6)	224 (77.2)	
Type of surgery			
Vascular	54 (2.3)	4 (1.4)	0.31
Cardiac	27 (1.2)	1 (0.3)	0.20
Orthopedic	21 (0.9)	1 (0.3)	0.33
Head and neck	6 (0.3)	0(0.0)	0.39
Gynecological	2 (0.1)	2 (0.7)	0.01
Gastrointestinal	46 (2)	177 (61)	<.01
Chronic co-morbidities			
At least one	1143 (49.0)	112 (38.6)	<.01
Respiratory	508 (21.8)	32 (11.0)	<.01
Cardiac	345 (14.8)	37 (12.8)	0.36
Renal	102 (4.4)	11 (3.8)	0.65
Immunodepression	338 (14.5)	34 (11.7)	0.20
Hepatic	157 (6.7)	20 (6.9)	0.91
Reason for mechanical ventilation			
Respiratory failure	965 (41.4)	36 (12.4)	<.01
Coma	436 (18.7)	10 (3.4)	<.01
Catecholamines	1284 (55.0)	196 (67.6)	<.01
SAPS II, median (IQR)^a^	53 [41; 66]	52 [41; 62]	0.28
SOFA, median (IQR)^b^	8 [5; 11]	7 [6; 10]	0.26
Nosocomial infection during the ICU stay			
VAP	806 (34.5)	56 (19.3)	<.01
Catheter-related	132 (5.7)	8 (2.8)	0.04
Urinary tract	63 (2.7)	3 (1.0)	0.09
Surgical site	34 (1.5)	21 (7.2)	<.01
Other site	14 (0.6)	2 (0.7)	0.85
Hospital			<.01
A	341 (14.6)	32 (11.0)	
B	79 (3.4)	36 (12.4)	
C	213 (9.1)	12 (4.1)	
D	141 (6.0)	10 (3.5)	
E	548 (23.5)	45 (15.5)	
F	124 (5.3)	9 (3.1)	
G	26 (1.1)	4 (1.4)	
H	713 (30.6)	139 (47.9)	
I	148 (6.3)	3 (1.0)	
Chest tube the first 2 days	255 (10.9)	25 (8.6)	0.23
Neuromuscular blockers the first 2 days	293 (12.6)	12 (4.1)	<.01
LOD score			<.01
<3	342 (14.7)	54 (18.6)	
3 to 5	758 (32.5)	114 (39.3)	
6 to 7	552 (23.7)	67 (23.1)	
>7	681 (29.2)	55 (19.0)	
ICU stay length in days, median (IQR)	16 [10; 27]	15 [9; 26]	0.49
Death in the ICU	747 (32)	81 (27.9)	0.16

IAI, intra-abdominal infection; IQR, interquartile range; BMI, body mass index; MOF, multiple organ failures; COPD, chronic obstructive pulmonary disease; SAPS II, Simplified Acute Physiology Score, version II; SOFA, Sequential Organ Failure Assessment; ICU, intensive care unit; VAP, ventilator-associated pneumonia

### Ventilator-associated pneumonia

**Fig 2:**
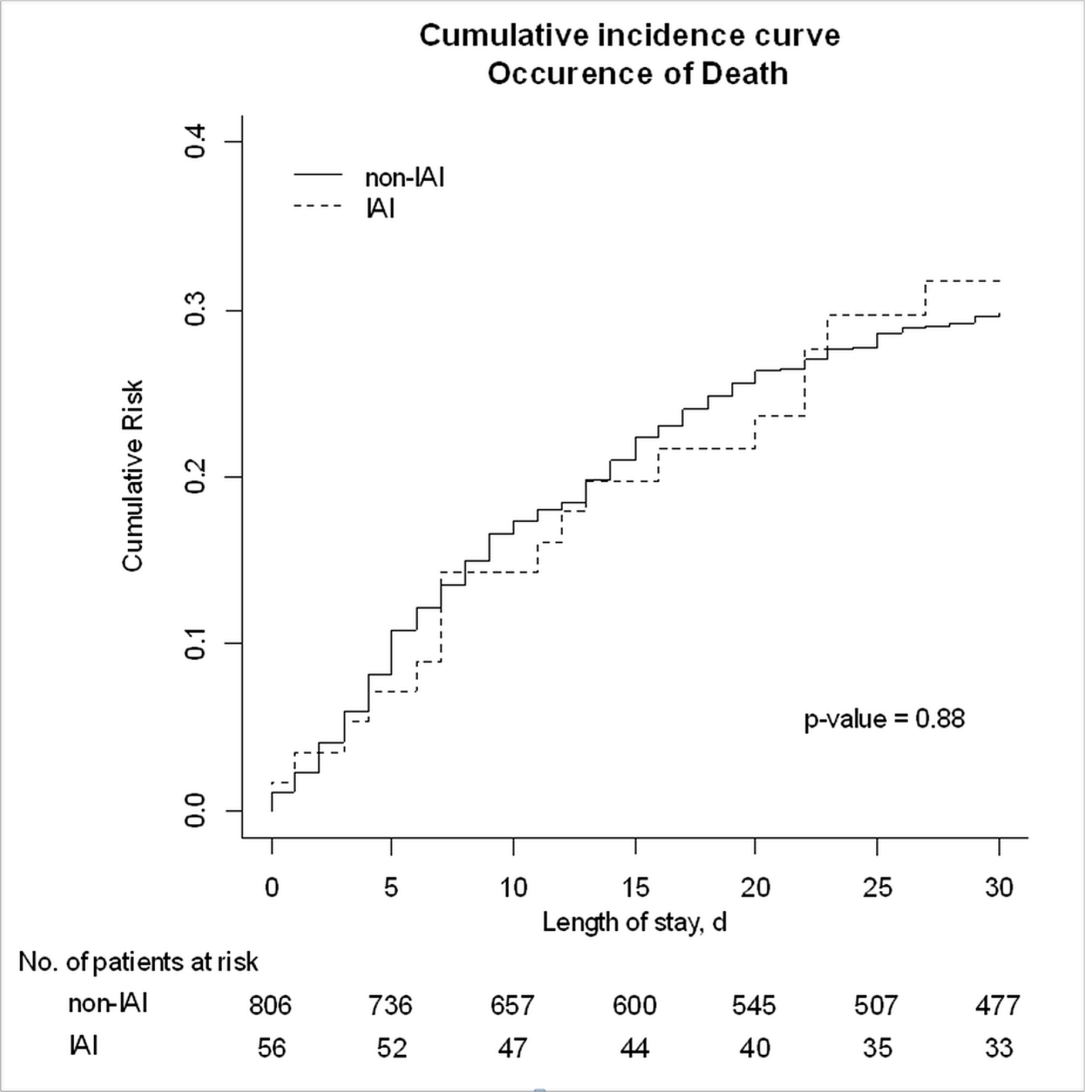
Occurrence of ventilator-associated pneumonia in patients admitted to the ICU for severe sepsis and/or septic shock related to intra-abdominal infections (IAIs) or to infections at other sites. Cumulative incidence plot.

VAP occurred in 56 (19.3%) patients in the IAI group and 806 (34.5%) in the non-IAI group (*P*<0.01) (Table 1). The median time to VAP was 5 [2; 10] in the non-IAI group and 10.5 [6.5; 17] in the IAI group (p < 0.01) (S3 Table). Both early and late VAP were less common in the IAI group (Fig 2). Frequency of early VAP (<72h) was 2.1% and 10.4% of patients in IAI and non-IAI group; frequency of late VAP was 17.2% and 24.1% respectively. In the Cox model taking presence of antibiotic therapy into account (Table 2), the difference remained significant (HR, 0.623; 95% CI: 0.463–0.837; *P* = 0.0017). Factors independently associated with an increase in VAP risk were male gender (HR, 1.29; 95%CI, 1.11–1.50; *P* = 0.0008), and smoking habits (HR, 1.22; 95%CI, 1.03–1.43; *P* = 0.02). The proportion of patients with VAP in whom the antibiotics used were effective against the recovered organisms was similar in the groups with and without IAI (28 [50.0%] and 339 [42.1%], respectively; *P* = 0.25).

**Table 2:** Result of the Cox model analysis of the impact of intra-abdominal infection on the subsequent risk of ventilator-associated pneumonia.

Parameter	DF	Parameter estimate	Std	Chi-Square	*P* value	HR	95%CI	
Antibiotic on the previous day	1	-0.25	0.07	11.68	**0.0006**	0.78	0.67	0.89
IAI	1	-0.47	0.15	9.84	**0.0017**	0.62	0.46	0.83
Hospital					**0.0001**			
A	1	-0.24	0.17	1.98	0.16	0.78	0.56	1.10
B	1	0.15	0.22	0.46	0.50	1.16	0.76	1.78
C	1	-0.01	0.19	0.00	0.95	0.99	0.68	1.43
D	1	0.32	0.19	2.78	0.10	1.38	0.94	2.02
E	1	-0.24	0.16	2.07	0.15	0.79	0.57	1.09
F	1	0.20	0.20	0.99	0.32	1.22	0.83	1.79
G	1	-0.49	0.37	1.77	0.18	0.61	0.30	1.26
H	1	-0.26	0.16	2.58	0.11	0.77	0.56	1.06
Male	1	0.26	0.08	11.32	**0.0008**	1.29	1.11	1.50
Smoking habits	1	0.20	0.08	5.65	**0.0174**	1.22	1.03	1.43
Chest tube the first 2 days	1	0.34	0.10	10.53	**0.0012**	1.40	1.14	1.72
Neuromuscular blockers the first 2 days	1	0.34	0.10	11.62	**0.0007**	1.41	1.16	1.72
Septic shock	1	-0.62	0.12	27.01	**<.0001**	0.54	0.42	0.68
Respiratory distress	1	-0.31	0.1	10.13	**0.0015**	0.73	0.60	0.89
COPD symptoms	1	-0.52	0.21	6.02	**0.0142**	0.59	0.39	0.90
LOD score					**0.0143**			
<3	1	0.20	0.11	3.24	0.07	1.23	0.98	1.53
3 to 5	1	-0.04	0.09	0.20	0.65	0.96	0.80	1.15
6 to 7	1	0.20	0.10	4.51	0.03	1.23	1.02	1.48
Chronic co-morbidities respiratory	1	-0.01	0.09	0.00	0.95	0.99	0.83	1.20
Age					0.81			
< 53	1	-0.07	0.11	0.38	0.53	0.94	0.76	1.15
53 to 65	1	-0.08	0.10	0.71	0.40	0.92	0.75	1.12
66 to 76	1	-0.01	0.10	0.01	**0.92**	0.99	0.82	1.20
MOF symptoms	1	-0.08	0.19	0.19	0.66	0.9	0.63	1.35
Coma symptoms	1	0.08	0.11	0.55	0.46	1.09	0.87	1.36

IAI, intra-abdominal infection; DF, degrees of freedom; HR, hazard ratio; 95%CI, 95% confidence interval; COPD, chronic obstructive pulmonary disease; LOD, Logistic Organ Dysfunction score

In the group with VAP, the subgroups with and without IAI differed regarding the reasons for ICU admission, admission category (medical, scheduled surgery, emergency surgery), and Glasgow Coma Scale score; they did not differ for age, smoking status, or number of co-morbidities (S3 Table). Time from ICU admission to VAP diagnosis was significantly longer in the IAI group (5.0 vs. 10.5 days; *P*<0.01).The distribution of the organisms responsible for VAP was not significantly different between the two groups. In the groups with and without IAI, *P*.*aeruginosa* was recovered in 345 (42.8%) and 24 (42.8%) VAP episodes, *Enterobacteriacae* in 264 (32.8%) and 19 (34.0%), and S. *aureus* in 215 (26.7%) and 17 (30.4%), respectively. No differences in antibiotic resistance were found between the two groups (S4 Table).

### Mortality

**Fig 3:**
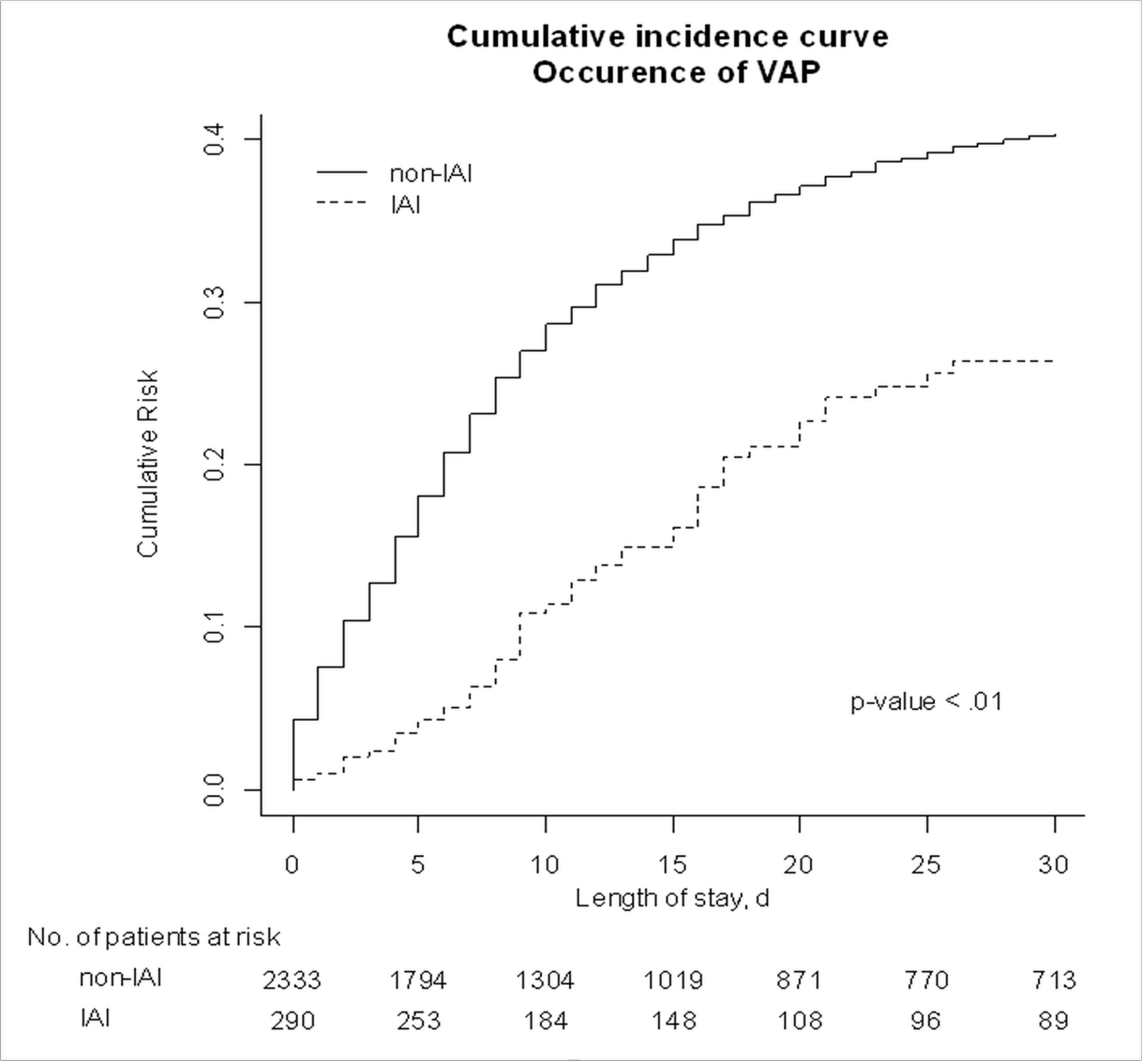
Cumulative incidence plot for death in patients with and without intra-abdominal infections (IAIs).

Crude ICU mortality was 27.9% (81 patients) in the IAI group and 32.0% (747) in the non-IAI group (*p* = 0.16) (Table 1). As expected, mortality was higher in the group with VAP. However, within the group with VAP, the cumulative incidence of death was not different between the IAI and non-IAI groups (Fig 3). Other factors associated with mortality were chronic co-morbidities such as hematological malignancies, cancer, or HIV infection and severity of the infection (septic shock, use of catecholamines, renal failure, impaired hematosis, and impaired hemostasis) (S5 Table).

## Discussion

In a large prospective cohort of ICU patients with severe sepsis and/or septic shock, IAI was associated with a decreased risk of VAP and longer time to VAP onset, compared to infections at other sites. Neither ICU mortality nor hospital mortality differed between the groups with and without IAI.

We are not aware of previous studies reporting a protective effect of IAI against VAP in patients with severe sepsis and/or septic shock. We chose to evaluate the potential influence of IAI on the occurrence of VAP because most animal models of sepsis-related immunodeficiency involve the induction of IAI. Our results are at variance with those of these animal studies, most of which suggest that IAI may increase the risk of VAP. First reading these results may seem surprising. However, human patients and animal models differ in many respects [24–26]. Experimental animals are typically young and devoid of chronic illnesses [25, 26] whereas our patients are older and have at least a chronic disease for about half of them in both groups. In addition, they show strong resistance to LPS [27]. Beside the technical parameters of achievement of infectious models, the time interval between the IAI and lung infection is of paramount importance [27]. In animal models, pathogens are instilled into the airways 2 to 24 hours after IAI induction [15, 16], which is a considerably shorter interval than the time from IAI to VAP in ICU patients. Thus, the animal models consist in co-infection rather than sequential infection. Interestingly, in a few studies involving a longer time to lung infection induction, bacterial clearance [13] and pneumonia control [28] were improved by previous infection. In addition, the size of the bacterial inoculum used to induce pneumonia may affect severity in animal models [15, 16, 25, 27]. Finally, the instillation of bacteria into the lungs does not replicate the pathogenic mechanism of VAP, which probably involves repeated bacterial microinhalations.

In a previous clinical study, intra-abdominal surgery was followed by a high incidence of hospital-acquired pneumonia with higher morbidity, longer hospital stays, and higher costs [29]. This study did not distinguish between patients with and without IAI and did not compare with other populations suffering from VAP. In our study in patients with severe sepsis and/or septic shock, VAP was less common in the group with IAI compared to the group with infections at other sites. The low overall incidence of VAP may be due to the use of quantitative distal samples for the diagnosis in most of our patients.

Several mechanisms may explain the decreased risk of VAP in our group with IAI. First, the administration of high antibiotic doses to treat IAI may protect against early VAP. An effect of this mechanism is unlikely in our study, however, as the patients in both groups had similar sepsis severity and received similar broad-spectrum antibiotics for similar durations. For the same reasons, the selection of reduced sensibility microbes due to the previous use of broad spectrum antibiotics is unlikely. Second, the paralytic ileus related to IAI often requires gastric tube placement to empty the stomach and the absence of enteral feeding, which may decrease the risk of inhalation and therefore the risk of VAP [22].

Third, the magnitude of the initial inflammatory response in the lung may affect the secondary antiinfectious response. The systemic inflammatory response that characterizes sepsis is associated with greater activation of pulmonary inflammatory processes in IAI than in other infections [30–32].

Fourth, next to these observations an important phenomenon that may be involved in pathophysiological mechanisms explaining the observed discrepancy between groups in our study is associated with the location (peritoneal) of the infection for many reasons: (a) the presence of endotoxemia [33]. It has been widely demonstrated that presence (and default of blood clearance) of LPS is associated with a compromised immunity leading to an increased risk of Gram-negative infection (which is the case in our study with 74.8 and 73.4% of VAP associated with Gram-negative bacteria in non-IAI and IAI group respectively) [34]. Peritonitis may be associated with a faster decrease in free and lipoproteins-bounded circulating LPS by early filtration of digestive venous blood by the liver (and notably Kupffer cells) which play a central role in LPS elimination [35] and surgical peritoneal cleaning reducing the amount of local bacterial fragments. (b) During intra-abdominal infection, more than during any other infection, the systemic response is associated with an inflammatory activation in the lung parenchyma [30–32] leading to the production by alveolar macrophages of proinflammatory cytokines and chemokines [32] favoring chemoattraction of monocytes [31, 36] and neutrophils [37, 38]. Systemic activated neutrophils can infiltrate lung capillary network before penetrating the parenchyma [38, 39]. Even after exposure to a first stimulation by LPS, as in the case of peritoneal infection, neutrophils still retain a proinflammatory phenotype and can respond to GM-CSF stimulation [40] which is present in large amount in the lung parenchyma [38]. Presence of alveolar macrophages, monocytes and activated neutrophils may protect the lung parenchyma from new pathogens colonization, reducing the bacterial inoculums and then preventing the occurrence of an early secondary VAP [41]. This notion is of particular interest as much as the first stimulation with a pathogen or any PAMP (pathogen associated molecular pattern) do not modify the ability of alveolar macrophages to produce a proinflammatory response during a second stimulation because of their resistance to endotoxin reprogramming [42, 43].

Finally, although patients with severe sepsis and IAI were at decreased risk for VAP, their risk of death was similar to that in the non-IAI group. The attributable mortality of VAP is about 6% [2] and our study was not powered to detect a significant difference in VAP-associated mortality. In surgical patients, particularly those with IAI, respiratory tract infections, most notably VAP, are usually associated with worse outcomes [44]. Earlier data obtained by our group suggest higher VAP mortality rates in surgical than nonsurgical patients [44]. The present study was not designed to assess attributable mortality, as we performed comparisons only within the group of patients with VAP.

Although we took a large amount of information into account and used highly effective statistical methods to correct for various biases, several limitations should be underlined.

First, a diagnostic criterion for VAP is the presence of chest radiograph abnormalities due to local inflammation. In patients with severe IAI but no pneumonia, such abnormalities are the rule, which may lead to delays in suspecting and diagnosing VAP. Another challenge when diagnosing VAP is that many patients with infections at non-intra-abdominal sites exhibit a variety of chest radiograph abnormalities, particularly when their first infection is pneumonia. Second, the diagnosis of VAP relied on different types of microbiological diagnosis techniques: quantitative cultures of tracheal aspirates or protected distal specimens (brush, telescopic catheter, or bronchoalveolar lavage), leading to difference in VAP incidence. Nonetheless such criteria correspond to the usual international guidelines, notably using clinical pulmonary infection score (CPIS) and its modified versions [45] leading us to consider the VAP diagnosis as relevant in our cohort. Third, we did not compare the incidence of VAP between patients with IAI and patients without infections at ICU admission. However, two such groups would differ markedly regarding numerous confounding factors, among which initial antibiotic therapy and duration of mechanical ventilation would be of paramount importance. Fourth, our non-IAI group was highly heterogeneous, as the types of infection varied widely. The number of infection-site subgroups would have been too large and the size of each subgroup too small for a valid subgroup analysis. In our point of view this is a very interesting point due to the opportunity to compare peritoneal infection which was largely studied in many animal models and as patient diseases to other infections that are usually not polymicrobial and do not involve such large amount of bacteria, LPS and do not benefit from liver immediate filtration. Finally, our database did not contain biological data (e.g., cytokine levels) or histological data relevant to the link between IAI and VAP, needing forthcoming studies to confirm our pathophysiological hypothesis.

## Conclusion

IAI as the cause of severe sepsis and/or septic shock seems associated with a lower risk and later occurrence of VAP compared to infections at other sites. This finding requires confirmation in other cohorts and warrants studies into potential pathophysiological mechanisms.

## Supporting Information

S1 Table csv raw data file in csv mode. (CSV)

S2 Table excel raw data file. (XLS)

S3 Table Characteristics of the patients in the subgroup with ventilator-associated pneumonia. IAI, intra-abdominal infection; IQR, interquartile range; BMI, body mass index; MOF, multiple organ failures; COPD, chronic obstructive pulmonary disease; SAPS II, Simplified Acute Physiology Score, version II; SOFA, Sequential Organ Failure Assessment; ICU, intensive care unit; VAP, ventilator-associated pneumonia.
(DOCX)

S4 Table Organisms responsible for ventilator-associated pneumonia. Bacterial resistance was defined as resistance to methicillin for *S*. *aureus*, resistance to ticarcillin and/or ceftazidime and/or imipenem for *P*. *aeruginosa*, and production of extended-spectrum beta-lactamase or hyperproduction of cephalosporinase for *Enterobacteriaceae*.
(DOCX)

S5 Table Result of the Cox model used to evaluate the impact of intra-abdominal infection on the risk of death after the development of ventilator-associated pneumonia. IAI, intra-abdominal infection; DF, degrees of freedom; Std: Standard; sHR: Hazard Ratio; 95%CI, 95% confidence interval; HIV, human immunodeficiency virus; DNR, do-not-resuscitate order.
(DOCX)
