# Supplementary material for: Decreased Risk of Ventilator-Associated Pneumonia in Sepsis Due to Intra-Abdominal Infection
Source: PLoS One. 2015 Sep 4;10(9):e0137262. doi: 10.1371/journal.pone.0137262 (PMC4560443; doi:10.1371/journal.pone.0137262)
Supplement: S3 Table — IAI, intra-abdominal infection; IQR, interquartile range; BMI, body mass index; MOF, multiple organ failures; COPD, chronic obstructive pulmonary disease; SAPS II, Simplified Acute Physiology Score, version II; SOFA, Sequential Organ Failure Assessment; ICU, intensive care unit; VAP, ventilator-associated pneumonia. (DOCX) [file pone.0137262.s003.docx]

**SI : Table 3. Characteristics of the patients in the subgroup with ventilatory-associated pneumonia**

|  | **N (%)** | | |  |
| --- | --- | --- | --- | --- |
| **Variables** | **Patients without IAI** | **Patients with IAI** | ***P* value** |  |
|  | **(n=806)** | **(n=56)** |  |  |
| Age in y, median (IQR) | 66 [53 ; 76] | 62.5 [54 ; 73] | 0.47 |  |
| Men | 557 (69.1) | 36 (64.3) | 0.45 |  |
| Current smoker | 227 (28.2) | 14 (25) | 0.61 |  |
| BMI, median (IQR) | 25.3 [22.5 ; 27.7] | 25.3 [22.1 ; 30.3] | 0.22 |  |
| Main reason for admission, n (%) |  |  |  |  |
| Coma | 161 (20) | 1 (1.8) | <.01 |  |
| Acute respiratory failure | 306 (38) | 11 (19.6) | <.01 |  |
| Septic shock | 103 (12.8) | 29 (51.8) | <.01 |  |
| Hemorrhagic shock | 34 (4.2) | 0(0.0) | 0.12 |  |
| Cardiogenic shock | 39 (4.8) | 0(0.0) | 0.09 |  |
| Other shock | 26 (3.2) | 1 (1.8) | 0.55 |  |
| MOF | 24 (3) | 8 (14.3) | <.01 |  |
| Trauma | 7 (0.9) | 0(0.0) | 0.48 |  |
| COPD | 30 (3.7) | 1 (1.8) | 0.45 |  |
| Admission category |  |  | <.01 |  |
| Medical | 631 (78.3) | 7 (12.5) |  |  |
| Scheduled surgery | 88 (10.9) | 5 (8.9) |  |  |
| Emergency surgery | 87 (10.8) | 44 (78.6) |  |  |
| Type of surgery |  |  |  |  |
| Orthopedic surgery | 5 (0.6) | 0(0.0) | 0.55 |  |
| Gynecologic surgery | 1 (0.1) | 0(0.0) | 0.79 |  |
| Head and neck surgery | 1 (0.1) | 0(0.0) | 0.79 |  |
| Heart surgery | 11 (1.4) | 0(0.0) | 0.38 |  |
| Vascular surgery | 22 (2.7) | 1 (1.8) | 0.67 |  |
| Chronic co-morbidities |  |  |  |  |
| At least one | 395 (49) | 25 (44.6) | 0.53 |  |
| Respiratory | 174 (21.6) | 6 (10.7) | 0.05 |  |
| Chronic cardiac failure | 119 (14.8) | 7 (12.5) | 0.64 |  |
| Chronic renal failure | 40 (5) | 3 (5.4) | 0.90 |  |
| Immunodepression | 111 (13.8) | 10 (17.9) | 0.39 |  |
| Hepatic | 60 (7.4) | 5 (8.9) | 0.68 |  |
| SAPS II, median (IQR)^a^ | 52 [40 ; 63] | 51.5 [38.5 ; 61] | 0.70 |  |
| SOFA, median (IQR)^b^ | 8 [5 ; 10] | 7 [5 ; 10] | 0.90 |  |
| Nosocomial infection during the ICU stay |  |  |  |  |
| Catheter-related | 6 (0.7) | 0(0.0) |  |  |
| Urinary tract | 5 (0.6) | 0 |  |  |
| Surgical site | 3 (0.4) | 3 (5.4) |  |  |
| Other nosocomial | 3 (0.4) | 1 (1.8) |  |  |
| Time to VAP in days, median (IQR) | 5 [2 ; 10] | 10.5 [6.5 ; 17] | <.01 |  |
| Proportion of antibiotic before VAP, median (IQR) | 0.87 [0.46 ; 1] | 1 [0.73 ; 1] | 0.02 |  |
| Effective antibiotic therapy within 24 hours | 339 (42.1) | 28 (50) | 0.25 | |
| ICU stay length in days, median (IQR) | 24 [15 ; 39] | 36 [22 ; 51] | <.01 | |

IAI, intra-abdominal infection; IQR, interquartile range; BMI, body mass index; MOF, multiple organ failures; COPD, chronic obstructive pulmonary disease; SAPS II, Simplified Acute Physiology Score, version II; SOFA, Sequential Organ Failure Assessment; ICU, intensive care unit; VAP, ventilator-associated pneumonia
