# Supplementary material for: Decreased Risk of Ventilator-Associated Pneumonia in Sepsis Due to Intra-Abdominal Infection
Source: PLoS One. 2015 Sep 4;10(9):e0137262. doi: 10.1371/journal.pone.0137262 (PMC4560443; doi:10.1371/journal.pone.0137262)
Supplement: S4 Table — Bacterial resistance was defined as resistance to methicillin for S. aureus, resistance to ticarcillin and/or ceftazidime and/or imipenem for P. aeruginosa, and production of extended-spectrum beta-lactamase or hyperproduction of cephalosporinase for Enterobacteriaceae. (DOCX) [file pone.0137262.s004.docx]

**SI : Table 4. Organisms responsible for ventilator-associated pneumonia**

|  | **No (%)** | | |
| --- | --- | --- | --- |
| **variable** | **Patients without IAI** | **Patients with IAI** | ***P* value** |
|  | **(n = 806)** | **(n = 56)** |  |
| *Pseudomonas aeruginosa* |  |  |  |
| Susceptible (%) | 271 (33.6) | 19 (33.9) | 0.963 |
| Resistant (%) | 74 (9.2) | 5 (8.9) | 0.950 |
| *Staphylococcus aureus* (%) |  |  |  |
| Susceptible | 157 (19.5) | 10 (17.9) | 0.767 |
| Resistant | 58 (7.2) | 7 (12.5) | 0.146 |
| *Enterobacteriaceae* (%) |  |  |  |
| Susceptible | 222 (27.5) | 16 (28.6) | 0.868 |
| Resistant | 42 (5.2) | 3 (5.4) | 0.962 |
| *Acinetobacter baumani*(%) |  |  |  |
| Susceptible | 19 (2.4) | 3 (5.4) | 0.169 |
| Resistant | 10 (1.2) | 1 (1.8) | 0.725 |

Bacterial resistance was defined as resistance to methicillin for*S. aureus*, resistance to ticarcillin and/or ceftazidime and/or imipenem for *P. aeruginosa*, and production of extended-spectrum beta-lactamase or hyperproduction of cephalosporinase for *Enterobacteriaceae*.
