# Supplementary material for: Decreased Risk of Ventilator-Associated Pneumonia in Sepsis Due to Intra-Abdominal Infection
Source: PLoS One. 2015 Sep 4;10(9):e0137262. doi: 10.1371/journal.pone.0137262 (PMC4560443; doi:10.1371/journal.pone.0137262)
Supplement: S5 Table — IAI, intra-abdominal infection; DF, degrees of freedom; Std: Standard; sHR: Hazard Ratio; 95%CI, 95% confidence interval; HIV, human immunodeficiency virus; DNR, do-not-resuscitate order. (DOCX) [file pone.0137262.s005.docx]

**SI: Table 5. Result of the Cox model used to evaluate the impact of intra-abdominal infection on the risk of death after the development of ventilator-associated pneumonia**

| **Parameter** | **DF** | **Parameter estimate** | **Std** | **Chi-Square** | ***P* value** | **sHR** | **95%CI** | |
| --- | --- | --- | --- | --- | --- | --- | --- | --- |
| **IAI** | 1 | 0.06936 | 0.26401 | 0.0690 | 0.7928 | 1.072 | 0.639 | 1.798 |
| Hospital |  |  |  |  |  |  |  |  |
| A | 1 | 0.21750 | 0.37461 | 0.3371 | 0.5615 | 1.243 | 0.596 | 2.590 |
| B | 1 | 0.73646 | 0.44002 | 2.8013 | 0.0942 | 2.089 | 0.882 | 4.948 |
| C | 1 | 0.30828 | 0.40220 | 0.5875 | 0.4434 | 1.361 | 0.619 | 2.994 |
| D | 1 | 0.76956 | 0.38084 | 4.0832 | 0.**0433** | 2.159 | 1.023 | 4.554 |
| E | 1 | 0.10041 | 0.34990 | 0.0824 | 0.7741 | 1.106 | 0.557 | 2.195 |
| F | 1 | 1.11537 | 0.39092 | 8.1405 | 0.**0043** | 3.051 | 1.418 | 6.564 |
| G | 1 | -0.58774 | 1.05651 | 0.3095 | 0.5780 | 0.556 | 0.070 | 4.406 |
| H | 1 | 0.84837 | 0.34654 | 5.9934 | 0.**0144** | 2.336 | 1.184 | 4.607 |
| Hematological malignancy | 1 | 0.66618 | 0.26733 | 6.2101 | **0.0127** | 1.947 | 1.153 | 3.288 |
| Solid cancer | 1 | 1.04351 | 0.25832 | 16.3182 | **<.0001** | 2.839 | 1.711 | 4.711 |
| HIV infection | 1 | 1.17704 | 0.43254 | 7.4052 | **0.0065** | 3.245 | 1.390 | 7.575 |
| Age, y |  |  |  |  |  |  |  |  |
| <53 | 1 | -0.71709 | 0.21330 | 11.3019 | **0.0008** | 0.488 | 0.321 | 0.742 |
| 53 to 65 | 1 | -0.73443 | 0.19795 | 13.7646 | **0.0002** | 0.480 | 0.325 | 0.707 |
| 66 to 75 | 1 | -0.11594 | 0.16800 | 0.4763 | 0.4901 | 0.891 | 0.641 | 1.238 |
| >75 |  |  |  |  |  | 1 |  |  |
| Obesity | 1 | -0.48953 | 0.22700 | 4.6505 | 0.**0310** | 0.613 | 0.393 | 0.956 |
| Septic shock | 1 | 0.57447 | 0.15548 | 13.6517 | 0.**0002** | 1.776 | 1.310 | 2.409 |
| Parenteral nutrition before VAP | 1 | -0.62958 | 0.28322 | 4.9413 | 0.**0262** | 0.533 | 0.306 | 0.928 |
| DNR before VAP | 1 | 1.47045 | 0.28100 | 27.3840 | <.**0001** | 4.351 | 2.509 | 7.547 |
| Dopamine at ICU admission >5 μg/Kg/min | 1 | 0.32460 | 0.14428 | 5.0615 | 0.**0245** | 1.383 | 1.043 | 1.836 |
| Bilirubin at ICU admission >59 mg/L | 1 | 0.82069 | 0.23327 | 12.3774 | 0.**0004** | 2.272 | 1.438 | 3.589 |
| Platelets at ICU admission <50·10^3^/mm^3^ | 1 | 0.73495 | 0.24215 | 9.2119 | 0.**0024** | 2.085 | 1.297 | 3.352 |
| Creatinine at ICU admission  >34 mg/L | 1 | 0.60353 | 0.16713 | 13.0398 | 0.**0003** | 1.829 | 1.318 | 2.537 |
| PaO_2_/FiO_2_<200mmHg | 1 | 0.37004 | 0.13459 | 7.5595 | 0.**0060** | 1.448 | 1.112 | 1.885 |

IAI, intra-abdominal infection; DF, degrees of freedom; Std: Standard; sHR: Hazard Ratio; 95%CI, 95% confidence interval; HIV, human immunodeficiency virus; DNR, do-not-resuscitate order
